# A novel splicing mutation in *SLC9A6* in a boy with Christianson syndrome

**DOI:** 10.1038/s41439-019-0046-x

**Published:** 2019-03-25

**Authors:** Daisuke Ieda, Ikumi Hori, Yuji Nakamura, Kei Ohashi, Yutaka Negishi, Ayako Hattori, Atsuko Arisaka, Setsuko Hasegawa, Shinji Saitoh

**Affiliations:** 1 Department of Pediatrics and Neonatology, Nagoya City University Graduate School of Medical Sciences, Nagoya, Japan; 2 Department of Pediatrics, Tokyo-Kita Medical Center, Tokyo, Japan; 3 Department of Pediatrics and Developmental Biology, Graduate School of Medical and Dental Sciences, Tokyo Medical and Dental University, Tokyo, Japan

**Keywords:** Paediatric neurological disorders, Genetics research

## Abstract

A loss of function mutation in *SLC9A6* (Xq26.3) is responsible for Christianson syndrome in males. We identified a novel splicing mutation (NM_006359.2:c.1141-8C>A) of *SLC9A6* in a seven-year-old boy with microcephaly, severe developmental delay, and intractable epilepsy. Functional analysis found multiple aberrant transcripts, none of which maintained the canonical open reading frame. Computer prediction tools, however, failed to detect all of the aberrant transcripts.

A loss of function mutation in the *SLC9A6* gene (Xq26.3) is responsible for Christianson syndrome (CS), which is characterized by severe global developmental delay, developmental regression, acquired microcephaly, intractable epilepsy, ataxia, ophthalmoplegia, and sometimes, death at a young age^1,2^. The clinical features of CS overlap with those of Angelman syndrome (AS), which is caused by a lack of expression of the maternally inherited *UBE3A* gene located on 15q11.2^3^. *SLC9A6* encodes the Na+/H+ exchanger protein NHE6. This protein regulates the luminal pH of early and recycling endosomes involved in the trafficking of proteins essential for structural and functional plasticity at glutamatergic synapses^4^. NHE6 has an important role in the growth of dendritic spines and the development of normal brain wiring^5^. Here, we identified a novel *SLC9A6* splicing mutation in a seven-year-old boy with microcephaly, severe developmental delay, and intractable epilepsy. To evaluate the mutation, we used various computer prediction tools as well as reverse transcription polymerase chain reaction (RT-PCR) and cloning to assess transcripts and confirm the pathogenicity of the mutation.

The case study, a seven-year-old Japanese boy, was born at term with a birth weight of 2978 g (−0.4 SD), length of 50.2 cm (+0.4 SD), and head circumference of 31.4 cm (−1.5 SD). His development delayed gradually, achieving head control at four months, sitting at nine months, and pulling to stand at two years. Currently, he cannot stand independently nor speak meaningful words. At 10 months of age, he developed intractable seizures of variable types: tonic-clonic convulsion, impairment of consciousness, focal seizure, and epileptic negative myoclonus. He was treated with multiple antiepileptic drugs that had insufficient therapeutic effects. An electroencephalography (EEG) performed at four years showed focal epileptic discharges with generalization in multiple foci (Fig. 1a). At four years of age, his weight was 14.5 kg (−0.7 SD), length was 102 cm (−0.1 SD), and head circumference was 46.2 cm (−2.6 SD), indicating microcephaly. Brain magnetic resonance imaging (MRI) (performed at 1, 2, and 4 years of age) and magnetic resonance spectroscopy (MRS) (performed at 4 years of age) showed no abnormal findings (Fig. 1b, c). At five years of age, we suspected the diagnosis of AS due to severe developmental delay, trunk ataxia, intractable seizures, microcephaly, and frequent smiling.

**Fig. 1:**
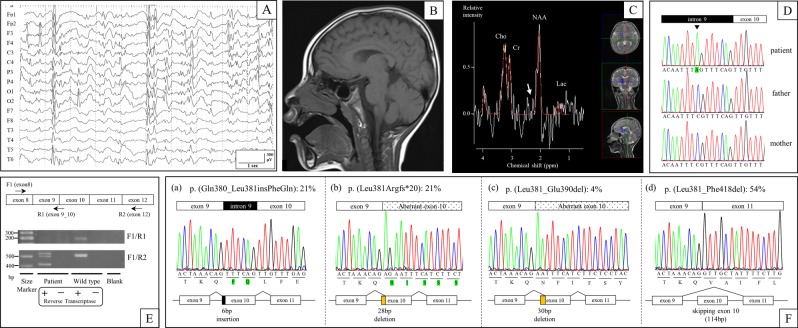
Clinical findings and genetic tests of the patient. **a** EEG performed at 4 years showed focal epileptic discharge with generalization in multiple foci. **b** Brain MRI (T1 weighted sagittal) performed at 4 years did not show any abnormal findings. **c** Brain MRS in the basal ganglia performed at 4 years did not show any abnormal glutamate/glutamine peaks (white arrow). **d** The patient carried a *de novo* hemizygous *SLC9A6* mutation (NM_006359.2:c.1141-8C>A) that was confirmed by Sanger sequencing. **e** RT-PCR analysis identified multiple aberrant transcripts but no canonical transcripts in the patient, while it identified only canonical transcripts in control DNA (wild type). **f**: Transcript variants in the patient. Twenty-one percent of transcripts included intronic 6-bp nucleotides (a), 21% excluded exonic 28-bp nucleotides (b), 4% excluded exonic 30-bp nucleotides (c), and 54% skipped exon 10 (d)

We first performed genetic tests, including fluorescent in situ hybridization (FISH), DNA methylation, and mutation analysis of the protein coding exons of *UBE3A* by Sanger sequencing, but all of the tests were normal. Next, we performed targeted next-generation sequencing with the Ion Torrent Personal Genome Machine system (Life Technologies, Carlsbad, California). An amplicon library of the target exons and flanking sequence was prepared with the use of an Ion AmpliSeq Custom Panel (Life Technologies), which included *UBE3A*, *SLC9A6*, *TCF4*, *MBD5*, *CDKL5*, *MECP2*, and *FOXG1*. Sequence analysis pipelines were established with use of the workflow in CLC Genomic Workbench 7.0 (CLC bio, Aarhus, Denmark). We identified a *de novo* hemizygous splicing mutation (c.1141-8C>A) in *SLC9A6* (NM_006359.2), which was confirmed by Sanger sequencing using the *SLC9A6*-intron 9-Fwd (5’-TCCACATTTGCTCCCTTCT-3’) and *SLC9A6*-exon 10-Rev (5’-ACCACATACTCAAAACCCAC-3’) primer pair (Fig. 1d). We predicted that the mutation affected RNA splicing because it resulted in a new AG acceptor site six nucleotides upstream of the canonical acceptor site of exon 10.

To evaluate the mutation, we used multiple computer prediction tools. CRYP-SKIP (http://cryp-skip.img.cas.cz/) provides an overall probability of cryptic splice-site activation (as opposed to exon skipping) termed *P*_CR-E_^6^. *P*_CR-E_ calculates a value between 0 and 1, and lower values favor exon skipping. The *P*_CR-E_ prediction score for *SLC9A6* (c.1141-8C>A) was 0.20, thus favoring exon skipping. Next, we used Alamut Visual software (version 2.10, Interactive Biosoftware, Rouen, France), which assesses genomic sequences (wild type and mutant) using five splicing prediction tools (SpliceSiteFinder-like, MaxEntScan, Neural Network Splice, GeneSplicer, and Human Splicing Finder) based on different algorithms^7^. All five algorithms predicted a strength reduction in the canonical acceptor site. The prediction scores for the aberrant acceptor site (six nucleotides upstream of the canonical acceptor site) increased with three algorithms, and the scores did not change at any point downstream of the canonical acceptor site (Table 1).

**Table 1:** In silico and functional splicing analysis from five prediction algorithms and in vitro RT-PCR analysis of mRNA transcripts

cDNA position^a^	SSF (0–100)		MaxEntScan (0–16)		NNSPLICE (0–1)		GeneSplicer (0–21)		HSF (0–100)		In vitro observed mRNA transcripts
	WT	MUT	WT	MUT	WT	MUT	WT	MUT	WT	MUT	
*Canonical splice site*											
c.1141	82.52	NE	8.27	1.58	0.74	NE	2.77	NE	88.42	85.82	0%
Cryptic splice site											
c.1141-6	NE	76.86	NE	5.30	NE	NE	NE	NE	NE	78.15	21%
c.1150	NE	NE	NE	NE	NE	NE	NE	NE	72.75	72.75	0%
c.1169	83.44	83.44	6.12	6.12	0.64	0.64	NE	NE	87.60	87.60	21%
c.1171	72.43	72.43	NE	NE	NE	NE	NE	NE	76.93	76.93	4%
c.1216	88.37	88.37	8.92	8.92	0.67	0.67	NE	NE	91.52	91.52	0%

*SSF* Splice Site Finder-like, *HSF* Human Splicing Finder, *WT* wild type, *MUT* mutant, *NE* not evaluated

^a^First nucleotide of the acceptor splice site

To confirm the RNA splicing results, we performed RT-PCR, cloning, and Sanger sequencing using total RNA from Epstein-Barr virus-induced lymphoblastoid cell lines established from peripheral leukocytes. RT-PCR using the *SLC9A6*-exon 8-Fwd1 (5′-ACCAAATTACGGGAGTTCCA-3′) and *SLC9A6*-exon 12-Rev2 (5′-CACCACCAAATACCCACAC-3′) primer pair revealed the presence of multiple transcripts (Fig. 1e). *SLC9A6* cDNA was then ligated into a TOPO cloning vector (Life Technologies) and transformed into TOP10 Competent Cells (Life Technologies), and 24 colonies were screened by extracting plasmid DNA using a QIAprep Spin Miniprep Kit (Qiagen, Hilden, Germany). Sanger sequencing of the plasmid clones identified four unique, aberrant transcripts but no canonical transcripts. Of the 24 cDNA transcripts screened, five (21%) had the six-nucleotide addition of the intronic sequence to the 5’ end of exon 10 that was predicted by our in silico analysis (Fig. 1f, transcript (a)). We also identified five (21%) transcripts and one (4%) transcript with 5’ exon 10 deletions of 28 and 30 nucleotides, respectively, as well as 13 transcripts (54%) with complete skipping of exon 10 (Fig. 1f, transcripts (b), (c), and (d)). Furthermore, we performed RT-PCR using the *SLC9A6*-exon 8-Fwd1 and *SLC9A6*-exon9_10-Rev1 (5′-GCTCAAACAACTGTTTAGTTCTA-3′) primer pair, which amplified only canonical transcripts, and it revealed canonical transcripts in control DNA but no amplification in that of the patient.

CS was first reported in 1999 in a Caucasian South African family with multiple affected males presenting with severe intellectual disability, mutism despite apparently normal hearing, intractable epilepsy, and limited life expectancy^1^. As some of the clinical features of CS are shared with AS, 1.8–5.5% patients with AS-like phenotypes have *SLC9A6* mutations^3,8^. The characteristic features that distinguish CS from AS are external ophthalmoplegia, developmental regression with loss of motor skills, progressive atrophy of the inferior cerebellar vermis, and an increased glutamine-glutamate peak in the basal ganglia on MRS^9^. Our patient, however, did not show any of these characteristic features at seven years of age. Pescosolido et al. reported that CS patients had regression in walking (57%), eating (14%), loss of few words/sounds (57%), eye contact/facial expressions (14%) and other fine/gross motor skills (14%) after a medical illness and/or seizure cluster^10^; therefore, we intend to follow our patient carefully.

Using target sequencing, we identified a *de novo* hemizygous intronic mutation (c.1141-8C>A) in *SLC9A6* (NM_006359.2), which resulted in a new AG acceptor site six nucleotides upstream of the canonical acceptor site of exon 10. In silico computer prediction analysis was performed prior to functional analysis of the mutation. CRYP-SKIP predicted the mutation would tend to cause exon skipping. Alamut visual predicted a decreased score for the canonical acceptor site of exon 10 in all five algorithms and an increased score for c.1141-6, which is adjacent to the aberrant AG acceptor site, in 3 of the 5 algorithms. To confirm the differential RNA splicing caused by the intronic mutation, we performed functional analyses using RT-PCR, cloning, and Sanger sequencing. We found multiple aberrant transcripts in *SLC9A6* involving exon 10, but no canonical transcripts were identified. Twenty-one percent of transcripts had the six-nucleotide addition of the intronic sequence to the 5′-end of exon 10, as predicted by our in silico analysis, which leads to a two-amino-acid insertion (p.(Gln380_Leu381insPheGln)) that we termed transcript (a). Transcripts (b) and (c) had 5′ exon 10 deletions of 28 and 30 nucleotides occurring in 21 and 4% of transcripts, respectively, leading to a p.(Leu381Argfs*20) frameshift in transcript (b) and a 10-amino-acid deletion (p.(Leu381_Glu390del)) in transcript (c). Fifty-four percent of transcripts had complete skipping of exon 10, termed transcript (d), due to a 114-bp deletion that led to a 38-amino-acid deletion (p.(Leu381_Phe418del)). Exon 10 in *SLC9A6* encodes part of the functional domain that interacts with angiotensin II type 2 receptor^11^. Transcripts (b) and (d) (frameshift mutation and single exon deletion, respectively) are likely to disrupt gene function. The functional consequences of transcripts (a) and (c) (small in-frame insertion/deletion) are unclear as they are also located in the functional domain and only correspond to 25% of transcripts. Since the phenotype of the patient is consistent with CS, we conclude that not enough functional transcripts of *SLC9A6* are being expressed, and the c.1141-8C>A mutation is pathogenic.

Comparing computer predictions to RNA transcript analysis, transcripts (a) and (d) were predicted by Alamut visual and CRYP-SKIP, respectively, but transcripts (b) and (c) were not predicted. Previous studies comparing the functional consequences of splice site mutations in *HR* (using CRYP-SKIP)^12^ and *MYBPC3*, *ACTC1*, and *SCN5A* (using Alamut analysis)^7^ concluded that prediction programs underestimate the impact of intronic mutations and that functional analyses, such as RT-PCR and minigene analysis, are necessary. In our experience, computer prediction tools predicted two of the four aberrant transcripts detected by RT-PCR, highlighting the need to develop more accurate computer prediction tools.

## Data Availability

The relevant data from this Data Report are hosted at the Human Genome Variation Database at 10.6084/m9.figshare.hgv.2543
